# Modelling field scale spatial variation in water run-off, soil moisture, N_2_O emissions and herbage biomass of a grazed pasture using the SPACSYS model

**DOI:** 10.1016/j.geoderma.2017.11.029

**Published:** 2018-04-01

**Authors:** Yi Liu, Yuefen Li, Paul Harris, Laura M. Cardenas, Robert M. Dunn, Hadewij Sint, Phil J. Murray, Michael R.F. Lee, Lianhai Wu

**Affiliations:** a Key Laboratory of Aquatic Botany and Watershed Ecology, Wuhan Botanical Garden, Chinese Academy of Sciences, Wuhan 430074, China; b Sustainable Agriculture Sciences, Rothamsted Research, North Wyke, Okehampton, Devon EX20 2SB, UK; c College of Earth Sciences, Jilin University, Changchun 130061, China; d School of Veterinary Science, University of Bristol, Langford, Somerset BS40 5DU, UK

**Keywords:** North Wyke Farm Platform, Spatial heterogeneity, Grid-to-grid simulation

## Abstract

In this study, we evaluated the ability of the SPACSYS model to simulate water run-off, soil moisture, N_2_O fluxes and grass growth using data generated from a field of the North Wyke Farm Platform. The field-scale model is adapted via a linked and grid-based approach (grid-to-grid) to account for not only temporal dynamics but also the within-field spatial variation in these key ecosystem indicators. Spatial variability in nutrient and water presence at the field-scale is a key source of uncertainty when quantifying nutrient cycling and water movement in an agricultural system. Results demonstrated that the new spatially distributed version of SPACSYS provided a worthy improvement in accuracy over the standard (single-point) version for biomass productivity. No difference in model prediction performance was observed for water run-off, reflecting the closed-system nature of this variable. Similarly, no difference in model prediction performance was found for N_2_O fluxes, but here the N_2_O predictions were noticeably poor in both cases. Further developmental work, informed by this study's findings, is proposed to improve model predictions for N_2_O. Soil moisture results with the spatially distributed version appeared promising but this promise could not be objectively verified.

## Highlights

• SPACSYS provided reasonable predictions of biomass, water dynamic and N_2_O fluxes.
• For runoff, single-point simulation performed better than grid-based simulation.
• The grid-based SPACSYS provided an improvement in spatial detail.
• The grid-based SPACSYS may account for hydrological processes of a sloping field.

## Graphical abstract

**Figure:**
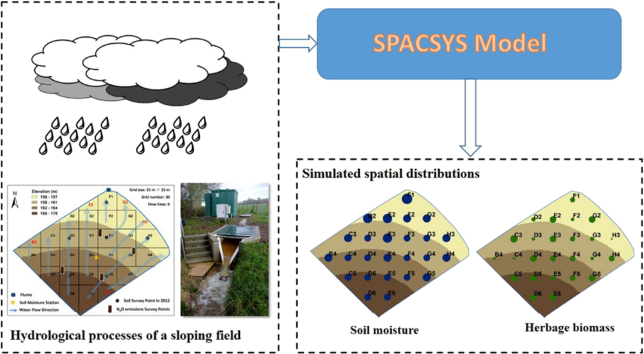

## 1 Introduction

Spatial variability in nutrient and water fluxes at the field scale is a key source of uncertainty when quantifying nutrient cycling and water movement in an agricultural system (Rowlings et al., 2012). Run-off production can be affected by spatial variation of the soil properties, the geology and the topography (Bell et al., 2009, Herbst et al., 2006), whilst variation in soil moisture directly affects the spatial characteristics of plant and ecosystem productivity (Li et al., 2017, Smith et al., 1997), soil carbon (C) and nitrogen (N) processes (Metcalfe et al., 2007, Prolingheuer et al., 2014, Rochette et al., 1991), microbial activity, chemical reaction rates and greenhouse gas (GHG) emissions (Konda et al., 2010). Thus, accounting for spatial effects in such processes is essential for understanding nutrient transformations in soil and losses to water and the air (DeSimone et al., 2010), and for water re-distribution (Li et al., 2016).

Process-based models can be an efficient tool for simulating spatial and temporal variations of a given process, including those for soil water and GHG emissions (Bell et al., 2009, Gala et al., 2011, Tian et al., 2010), and provide a useful alternative to resource-intensive field experiments (Jones et al., 2017). Commonly, a grid-based (grid-to-grid) modelling approach is adopted (Bell et al., 2007, Rathjens et al., 2015), where the resolution of the spatial discretization is commonly key to its success (von Gunten et al., 2014, Yu et al., 2011). For example, Dai et al. (2012) found the grid resolution (in this case, coupled with hydrological and biogeochemical models) to play a crucial role in reducing uncertainty of the simulated GHG emissions from wetland watersheds. However, there are drawbacks to the use of spatially distributed process-based models, as by design, they tend to require a large amount of measured data for their evaluation (Kawamura et al., 2011).

SPACSYS (Soil-Plant-Atmosphere Continuum System) is a process-based model, that can simulate plant growth and development, soil N and C cycling, soil water movement and heat transformation at the field scale (Wu et al., 2007). At this scale, it has been used to predict GHG emissions, soil C and N stocks, and crop yield without considering spatial variations in both parameters and outputs (Abalos et al., 2016, Perego et al., 2016, Wu et al., 2015, Wu et al., 2016a, Zhang et al., 2016). This standard (single-point) application of SPACSYS might not cause large uncertainties in simulation outputs for processes that can be assumed to be spatially homogeneous at the field scale. However, this might be seen as a highly naïve assumption, as all (single-point) input parameters for SPACSYS would unlikely be representative of spatial variations in soil physical and chemical properties. These are inherently spatial processes that are often driven by changes in topography and associated water flow directions. The problem relates to what hydrologists refer to as the ‘upscaling problem’, where it is not clear how to aggregate (or average) spatially distributed data, to then use as a single model input. A related problem is known as the ‘modifiable areal unit problem’ in geography (Openshaw, 1984), where depending on the level of aggregation used, different relationships between spatial processes will result, often masking complex non-linearities. Thus, except for cases where input data is very scarce, and an arithmetic mean provides the only pragmatic option, it is preferable to apply a spatially distributed model. In this respect, the aim of this study is to adapt SPACSYS to a spatial form, where hydrological processes coupled with *multiple sets* of spatially-indexed input parameters are used in order to improve the simulation accuracy of soil moisture, water and N_2_O fluxes, and biomass productivity.

The North Wyke Farm Platform (NWFP) is a farm-scale research platform for grassland-based beef and sheep production that was established in 2010 in southwest England (Orr et al., 2016). The NWFP provides three farming systems (farmlets): (i) permanent pasture (not reseeded for 10 + years), (ii) grass (perennial ryegrass) and white clover leys and (iii) an improved monoculture grass sward with planned regular (3–5 years) reseeding. The NWFP is typical of lowland grassland systems in western regions of the UK. Each farmlet consists of five hydrologically isolated sub-catchments each comprising approximately 21 ha. Data are collected regularly, for each sub-catchment, on water run-off and chemistry, precipitation and soil moisture, with GHG emissions, soil nutrients and soil biology being collected occasionally, all of which is coupled with detailed farm management records. As would be expected, the soils of the NWFP display strong spatial heterogeneity and should not be taken as homogeneous within a field (Granger et al., 2017, Harris et al., 2016, Peukert et al., 2016).

Runoff, soil moisture, GHGs and plant biomass are frequently cited as major indicators for understanding the soil-plant-atmosphere ecosystem. Thus, this study has a focus on simulating these particular indicators across one (permanent pasture) field of the NWFP using standard and spatially distributed versions of the SPACSYS model. In addition to a potential for improving simulation accuracy, the outputs of the spatially distributed version can complement and enhance ground-based field surveys that are often costly to conduct, especially with any temporal regularity. We adopt a grid-to-grid approach for the spatial adaptation, which accounts for the spatial characteristics in the field's hydrological processes, and in turn, the field's soil and topographic properties for run-off production and nutrient cycling.

The aim for this study is to demonstrate the potential of the spatially distributed version of the SPACSYS model to provide more accurate simulations than that found in the standard version, which in turn should provide an improved understanding of the spatial dynamics of C and N processes. In particular, this study will: (1) specify, validate and compare the two versions of SPACSYS using measured water runoff, soil moisture, soil N_2_O emissions and herbage biomass; and (2) apply (grid-to-grid) SPACSYS to simulate those indicators that display promising validation results in (1), at the within-field scale across annual time periods.

## 2 Materials and methods

### 2.1 Field site description

The NWFP is located in the southwest of England (50°46′10″N, 3°54′05″W), whose objective is to act as a test-bed for agricultural models, making particular use of its fine-resolution temporal data (e.g. water flow and chemistry data). The monitoring system of the NWFP is unique in both scale and scope for a managed land-based capability. It brings together several technologies that enable the effect of temperate grassland farming systems to be studied in detail. Rigorous data management, quality control and validation provide the basis for accurate assessments of the losses and gains between increased agricultural production and the provision of ecosystem services, at any given time interval, for each of the three farmlets. Data generated from the NWFP are freely available from http://www.rothamsted.ac.uk/farmplatform.

**Fig. 1:**
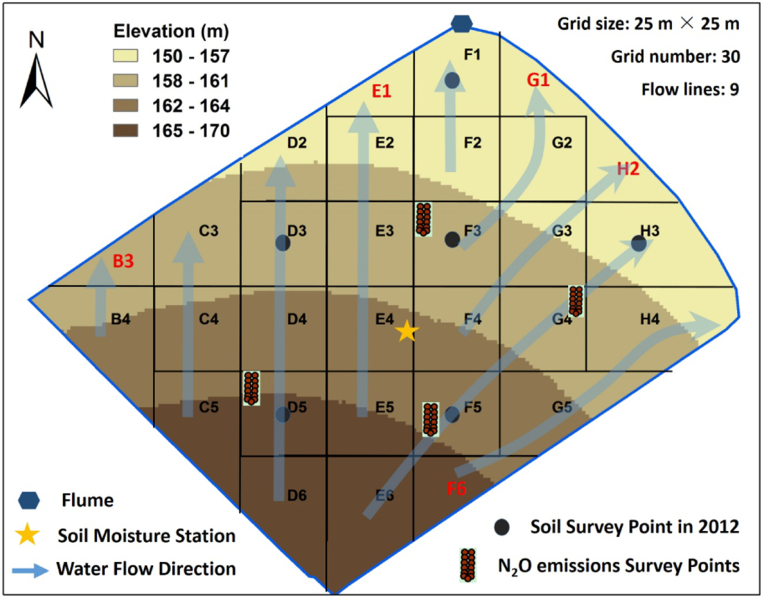
The NWFP study field with observation points and simulation grids, together with key features for runoff production and routing scheme for the grid-to-grid SPACSYS model. (For interpretation of the references to colour in this figure, the reader is referred to the web version of this article.)

To evaluate the SPACSYS models, measured data from one permanent pasture sub-catchment were used - in this case a single field, called Dairy North (1.78 ha in size) (Fig. 1). This field slopes downwards from a south to north direction, to a water flume in its northern corner that captures the field's water run-off, aided via a system of French drains that was constructed along the edges of the sub-catchment (800-mm deep trenches that contain a perforated drainage pipe backfilled to the surface with 20–50 mm clean granite, carbonate-free, stone chips).

### 2.2 The SPACSYS model: description and modification

The SPACSYS model is a process-based model with a daily time step. Briefly, the main processes concerning plant growth are assimilation, respiration, water and N uptake, partitioning of photosynthate and N, N-fixation for legume plants and root growth. The Richards equation for water potential is used to simulate water and heat fluxes. Water flow from the soil profile to a drainage pipe is estimated when the ground water table is above the bottom level of the pipe and the soil below the ground water table is saturated. Moreover, N cycling coupled with C cycling in the model covers the transformation processes for organic matter and inorganic N plus a biological-based component for the denitrification process that can estimate N gaseous emissions. Within a given simulation, N_2_O is emitted during nitrification and denitrification. Nitrification rate is estimated based on ammonium and nitrate contents in the soil, soil temperature and moisture, and soil acidity. Denitrification is simulated through an approach based on microbial activity (Wu et al., 2015). Gas emission rates are proportional to NO, N_2_O and N_2_ contents near the soil surface and adjusted by a reduction function which depends on soil gas diffusion rate, that in turn depends on air-filled porosity, soil temperature and soil texture. Therefore, SPACSYS incorporates functions for simulating plant development and growth, water and N uptake, soil C and N cycling, GHG (e.g. CO_2_ and N_2_O) emissions, and water redistribution. Details of the SPACSYS model have been reported elsewhere (Wu et al., 2007, Wu et al., 2015, Wu et al., 2016a), thus only those modifications related to this study, are presented here.

The standard version of the SPACSYS model is only able to simulate isolated fields (single-point) which does not consider topographic or spatial connectivity between fields or within the field itself. In the single-point version, water flow is assumed as being homogeneous across the field and only a single set of input parameters is required to characterise the whole field, i.e. it could be viewed as an input parameter set relating only to the centre grid cell E4 in Fig. 1. For this study, our concern is within-field variability, where we consider the water flow (i.e. topographical) characteristics as depicted in Fig. 1. Here the SPACSYS model is adapted to a grid-based form (grid-to-grid), where the topographical inter-connections of the field's water flow and nutrient pathways are accounted for. At each time step, the simulations start from those grid-cells that have no upstream input. Soil water and nutrients out of a grid-cell through surface runoff and drainage flow is passed to its recipient grid-cell as input before the simulation for the grid-cell starts at the time step.

For the spatially distributed simulation framework, 30 grid-cells within the field were taken via points on a 25 × 25 m grid. This grid resolution is chosen as it matches the highest spatial resolution of the model evaluation data (i.e. the biomass data, detailed below); it is also a common grid resolution for many sampling campaigns conducted in this NWFP field. The runoff-production schemes for the grid-to-grid formulation required gridded estimates of average terrain slope in each grid-cell, as implemented in ESRI ArcGIS (http://www.esri.com). To simplify the simulation conditions, it was assumed that each grid-cell can drain in only one of eight possible flow directions and each grid-cell can receive only one upstream grid cell, as detailed in Fig. 1. In total, there were nine water flow lines. All water simulations from each flow line are summed to compare with the single measured water flow at the flume. The field or sub-catchment is a closed system and in this respect the grid-to-grid simulations only need to be summed.

### 2.3 Measured data for model inputs

Soil physical and chemical properties of the field (bulk density (BD), soil organic matter (SOM), total organic carbon (TOC), total organic nitrogen (TON) and pH), used as an input to SPACSYS, were based on a NWFP-wide soil survey conducted in 2012 (Peukert et al., 2016) where samples in Dairy North were only taken on a coarse 50 m grid which yielded only six samples (Fig. 1). These six samples are co-located with six points of the 25 × 25m grid for this study. For the spatially distributed SPACSYS model, the five measured properties at these six sites were assigned to the remaining 24 of 30 sites, following simple nearest neighbour rules (see Fig. 1 and Table 1), i.e. without any form of distance weighted spatial interpolation, but still assuming a (user-defined) level of spatial dependence. It is unfortunate that the spatial resolution of this measured data is so coarse, where ideally the soils data would have been measured at all 30 sites. For the standard SPACSYS model, the simple arithmetic mean of BD, SOM, TOC, TON and pH was used in each case. Thus, the standard model assumes no within-field variability of these soils characteristics. Other soil physical properties were estimated with pedotransfer functions (PTFs) (Saxton et al., 1986) based on soil texture presented in the soil survey (Harrod and Hogan, 2008). Soil profiles were divided into eight layers for each grid with the thickness from the top to the bottom: 0.1, 0.1, 0.1, 0.4, 0.6, 0.15 and 0.2 m.

**Table 1:** Soil physical and chemical properties at 6 points in Dairy North measured in 2012.

Sample ID	Simulation IDs applied to	BD g cm^− 3^	SOM g kg^− 1^	TOC g kg^− 1^	TON g kg^− 1^	pH
1	E1, F1, G1	0.94	117.40	53.49	6.18	5.76
2	D2, B3, C3, D3	0.88	129.80	60.03	6.64	5.81
3	E2, F2, G2, E3, F3, G3	0.94	126.93	65.89	6.85	5.86
4	H2, H3, H4	0.92	117.71	57.97	6.13	5.91
5	B4, C4, D4, C5, D5, D6	0.94	129.56	57.05	6.74	5.68
6	E4, F4, G4, E5, F5, G5, E6, F6	0.89	126.24	58.39	6.78	5.69

**Fig. 2:**
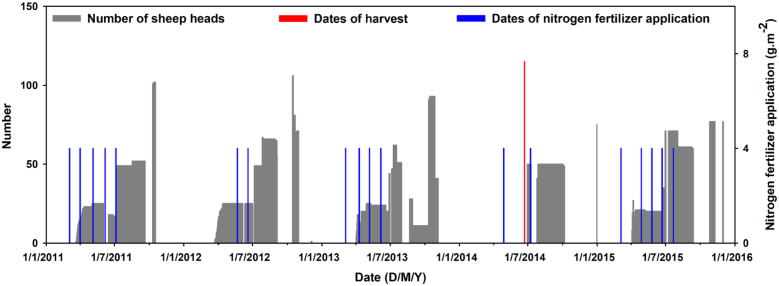
Number of sheep heads, field management, and fertilizer applications in the field.

Both versions of SPACSYS, use the same field and grass management (e.g. fertilizer application dates, start/end of grazing periods, livestock density, and cutting dates) inputs, as shown in Fig. 2. These data were interpreted from the extensive farm records for the NWFP experiment. Daily grass/forage intake and excretion of animals in the field were quantified following Wu et al. (2016a). Daily meteorological data over the simulation period were obtained from an automatic official MET office weather station together with a collocated automatic NWFP weather station, where the latter could only provide data from 2013 onwards. Both weather stations are 450 m from the centre of the study field and thus the meteorological data are safely considered representative of it. Again, both versions of SPACSYS used the same meteorological data. Thus, in summary, only the soil physical and chemical properties of the field varied spatially as inputs.

### 2.4 Measured data for model evaluation

For model evaluation, the two versions of SPACSYS were run for the selected periods between October 2011 to December 2015, depending on data availability at the time of this research. The following data sets were used to evaluate the resultant simulations: temporal-only water flux data, temporal-only soil moisture data, (intermittent) spatio-temporal N_2_O emissions and a relatively coarse-resolution spatial-only data set of herbage biomass. Thus, at this stage it needs to be stressed that model evaluation is often somewhat compromised by sparse temporal or sparse spatial availability of the measured data. This tends to reflect a resource issue, where the ideal spatio-temporal data sets are too expensive to collect.

Data for water fluxes and soil moisture are at a high 15-minute resolution. Water fluxes from the field are measured through a combination of primary and secondary flow devices at the field's flume site (see Fig. 1). The primary device is an H-type flume (TRACOM Inc., Georgia, USA) where flow rate can be determined by a known relationship (rating curve) against the height of the water at a single specific location in the flume. A secondary flow measurement device (4230 bubbler flow meter, Teledyne ISCO, New England, USA) is then used to measure the water height within the flume and convert this measurement to a flow or discharge rate. Soil moisture is measured at a centrally-located soil moisture station of the study field (see Fig. 1), via a combination soil moisture probe which measures soil moisture through capacitance at depths of 10, 20 and 30 cm. Here we only used the 10 cm data because the soil moisture was more sensitive in surface soil compared with subsoil, and more importantly, measured data at 20 and 30 cm was known to be unreliable. Because the SPACSYS model currently only generates daily outputs, soil moisture and water flux data sets were processed into a daily form. This temporal resolution is not ideal as time steps less than 1 h should be preferred (Mertens et al., 2002), but is not considered a serious problem for a model comparison focused study, such as that presented here.

For N_2_O emissions, daily data from June to November 2015 was measured using the Li-Cor Automated Soil Gas Flux System (LI-COR Inc., Nebraska, USA) with a set of 12 chambers that took gas samples sequentially. To include the effect of grazing in the measurements, the full 12-chamber set was moved to one of four pre-set locations in the field (see Fig. 1) every two weeks, apart from the last move, where it remained until the end of the sampling campaign. Once the chambers were moved from a pre-set location, sheep grazed that area until the next time the chambers were brought back. Opaque long-term Licor chambers (LI8100-104) were used to measure gas fluxes, with the 12 chambers connected to a Licor LI8100A gas analyser via a LI8150 multiplexer. In addition, an INNOVA 1412 photoacoustic gas monitor was used to measure N_2_O, being plumbed in parallel to the LI8100A exhaust line to the multiplexer. Measurement frequency from the Licor analyser was one per second, and approximately 120 s from the INNOVA analyser. Each chamber was closed for 16 min to allow for sufficient measurements of N_2_O concentrations to be able to estimate the emissions, and to leave enough time before and after each closure for flushing the lines. It took under 4 h to go around all the 12 chambers, which means that in 24 h each chamber measured six times. The N_2_O data were pre-processed into daily fluxes using the SoilFluxPro software from Licor. Observe there is no model evaluation opportunity for soil moisture at any of the four N_2_O data locations, as they do not collocate with the soil moisture station (Fig. 1). This is unfortunate given that antecedent soil moisture history can have an effect on N_2_O emissions (Bergstermann et al., 2011).

Ground herbage biomass was available in a spatial form at 25 of the 30 sites of the 25 × 25 m grid, i.e. each grid-cell centre, excluding five sites on the field's boundaries, marked in red in Fig. 1. These data were collected as part of a larger sampling campaign conducted on 9th June 2015 using clippers and the dry-weight of each biomass sample was recorded.

### 2.5 Statistical analyses for model evaluation

To evaluate the performance of the single-point and grid-to-grid SPACSYS models, a subset of the statistical diagnostics suggested by Smith et al. (1997) were used to compare the simulation outputs against the measured data. Here we define the *error* as *measured minus simulated* data. For the temporal-only evaluations (all except herbage biomass), standard goodness of fit model diagnostics of: (a) mean error (ME, over-prediction, −∞ < ME < +∞, under-prediction), (b) root mean squared error (RMSE, 0 (optimal) ≤ RMSE < +∞), (c) mean absolute error (MAE, optimum, 0 (optimal) ≤ MAE < +∞), (d) relative error (RE), (e) modelling efficiency (EF, −∞ < EF < 1 (optimal), where negative EF values indicate that the mean of the measured data is a better predictor then the model results (Smith et al., 1997)), (f) correlation coefficient (*r*) between the measured and simulated data (− 1 ≤ *r* ≤ 1) and (g) coefficient of determination (CD) were used for model evaluation. For the spatial-only evaluation (i.e. herbage biomass), the *R*^2^ from a linear regression fit is used. Observe that although RMSE and MAE relay similar accuracy characteristics, MAE is more resistant to high outlying errors.

## 3 Results

### 3.1 Model evaluation

#### 3.1.1 Water fluxes

**Fig. 3:**
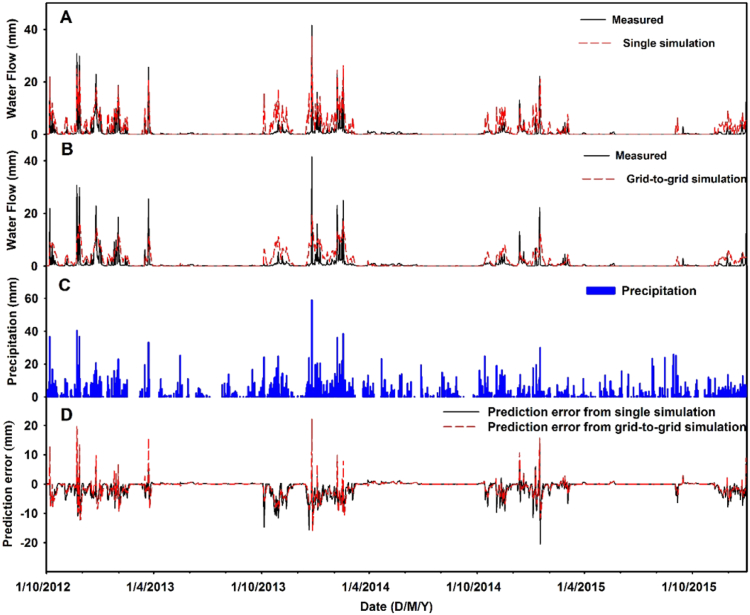
Comparison of measured and simulated water fluxes for the single-point (A) and grid-to-grid simulations (B), together with precipitation (C), and prediction errors (D).

Daily simulated water fluxes from the single-point and grid-to-grid models were compared with the measured data at the water flume for the period of 1/10/2012 to 31/12/2015 (Panels A and B of Fig. 3). The corresponding prediction errors (*measured minus simulated*) are given in Panel D of Fig. 3. The overall accuracy of each model's performance is given by the statistical diagnostics (Table 2). In general, the simulations for water flux appear broadly accurate from both single-point and grid-to-grid simulations, where most observed peak flow events were identified. However, both models under-predict the water fluxes to some degree during high rainfall periods, whilst both models over-predict at low flows. This behaviour is much more apparent for the grid-to-grid simulation, especially with respect to under-prediction at high flows. The grid-to-grid simulations are also smoother than the single-point simulations (i.e. the dynamic simulated curve of water fluxes showed a smaller amplitude with the grid-to-grid simulation). Overall this implies that the implementation of the grid-to-grid simulation has little effect on the performance of SPACSYS on modelling the dynamics of water flux, resulting in a low sensitivity and weak identifiability. The statistical diagnostics (Table 2), confirms the tendency for the single-point simulation to describe the measured data marginally better than the grid-to-grid simulation.

**Table 2:** Statistical analysis of SPACSYS's performance on dynamics of water fluxes for the single-point and grid-to-grid simulations.

Criteria	Single-point simulation	Grid-to-grid simulation
ME	− 0.98	− 0.91
RMSE	2.62	2.75
MAE	1.26	1.32
RE	− 865.12	− 1283.11
EF	0.50	0.28
*r*	0.75	0.59
CD	0.93	0.80

#### 3.1.2 Soil moisture

**Fig. 4:**
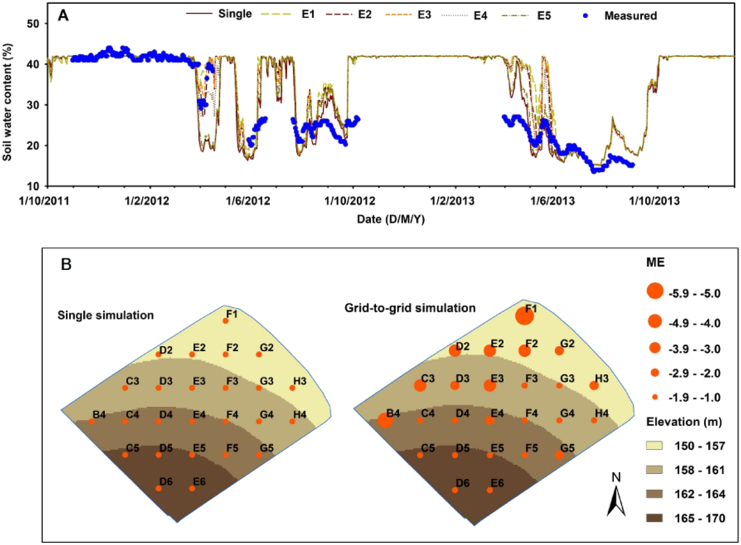
Temporal comparison of measured (circle) and simulated (line) soil moisture from the single-point and grid-to-grid models (A) and spatial comparison of ME values from the single-point and grid-to-grid models (B). For the spatial presentation, the results at the five incomplete grid cells (from Fig. 1) are not shown.

Daily simulations of soil moisture generated from the two versions of the SPACSYS model are compared to measured daily data for the two-year period 1st October 2011 to 30th September 2013 in terms of the temporal patterns (Panel A in Fig. 4). Simulations from a transect from E5 to E1 from the grid-to-grid model are shown that traverses key changes in topography, and is assumed to similarly traverse key changes in soil moisture. Given the nature of the field's topography, it is expected that soil moisture simulations would be generally lower in the southern part of the field, which should be the driest part, whilst simulations would be generally higher in the northern part of the field, which should be the wettest. The statistical accuracy of the grid-to-grid simulations at grid points E4 and F4 are shown in comparison to the accuracy range for all the grid points from the grid-to-grid model, and to the single-point model (Table 3). Thus, on average, simulations from the single-point model perform similarly to those outputted from the grid-to-grid model for grid points E4 and F4 (i.e. the grid points closest to the soil moisture station (see Fig. 1)). In an overall sense, the soil moisture simulations all tend to over-predict, as the MEs (calculated from measured soil moisture at the central location minus each simulation in turn) are always negative in Table 3. The broad temporal trends in the measured soil moisture are accounted for with the simulations, especially when the soil is approaching saturation. However, there are large discrepancies between the measured and simulated soil moisture data during dry periods. Temporally, these simulations also display both under- and over-predictions in relation to the centrally-located measurements. Here, over-prediction from the grid-to-grid model tends to be much lower in the southern, driest part of the field (e.g., by grid point E5), whilst much higher in the northern, wettest part of the field (e.g., by grid point E1). Therefore, it is assumed that the simulation with the grid-to-grid model can quantify spatial variation of soil moisture, at least in a relative sense. Of course, this finding is not proven as we have no spatially measured soil moisture to objectively evaluate the grid-to-grid simulations against, and in this respect the spatial ME's in Panel B of Fig. 4 are only given for a visual impression of the potential of the grid-to-grid SPACSYS model. Again, all spatial ME's are negative, reflecting a consistent over-prediction of soil moisture.

**Table 3:** Statistical analysis of SPACSYS's performance on dynamics of soil moisture for all grid-to-grid simulations, grid-to-grid simulations at grid points E4 and F4 only, and the single-point simulation.

Criteria	Grid-to-grid simulation	E4	F4	Single-point simulation
ME	− 8.09 to − 1.04	− 2.74	− 1.06	− 1.01
RMSE	5.90–11.20	6.29	5.95	5.93
MAE	3.77–8.44	4.13	3.81	3.79
RE	− 39.79 to − 9.14	− 15.43	− 11.18	− 11.67
EF	0.28–0.50	0.44	0.47	0.47
*r*	0.65–0.85	0.85	0.84	0.84
CD	0.80–0.93	0.83	0.84	0.84

#### 3.1.3 N_2_O emissions

**Fig. 5:**
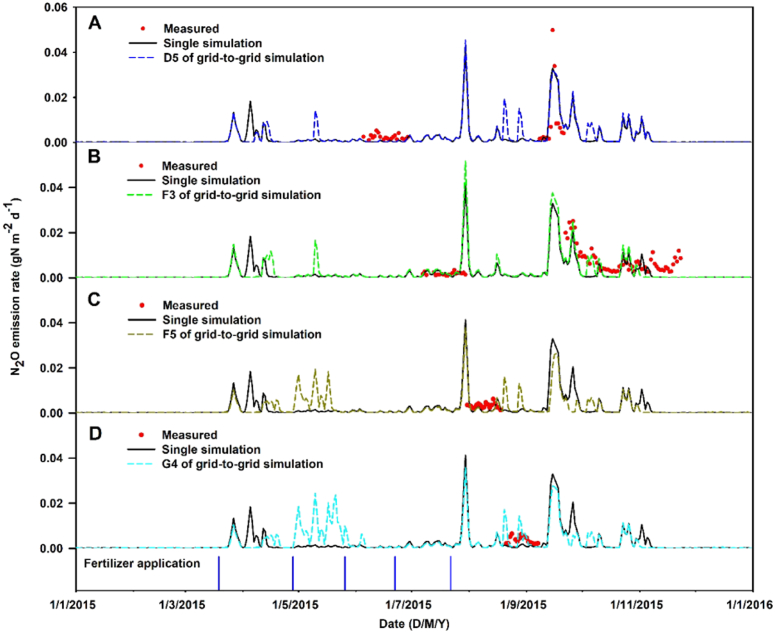
Comparison of measured (circle) and simulated (line) N_2_O emissions from the single-point and grid-to-grid simulations at grid points D5, F3, F5 and G4 (panels A to D, respectively). The vertical lines at the bottom indicate the dates when fertilizer was applied.

The single-point and grid-to-grid simulated daily N_2_O emissions (for grid points D5, F3, F5 and G4 only – which are closest to the four N_2_O sampling locations, see Fig. 1) are temporally compared to measured N_2_O data (Fig. 5). As N_2_O emissions were measured at each of four locations in an interval rota, the measured N_2_O data is somewhat compromised both spatially and temporally, where the N_2_O simulations are never a full match to that measured in both space and time. Given this caveat, the daily simulations from both versions of SPACSYS do not appear to capture the seasonal fluctuations of N_2_O emissions well, although the N_2_O emission peak during 19–28th September is captured. Furthermore, both simulations, suggested N_2_O emission peaks when they did not exist in the measurements, for example, the simulated N_2_O emission peaks on 30th July. Tentatively, the grid-to-grid simulations appear to better account for fertilizer applications than the single-point simulations. Table 4 provides the statistical accuracy of the two SPACSYS models for predicting N_2_O at grid points D5, F3, F5 and G4, where both models perform in a similar manner. The expected improvement with the grid-to-grid model is not apparent and is a likely reflection of having only four sampling sites available, which are also a poor representation of the field. The small sample evaluation sizes at grid points F5 and G4 are also a likely mitigating factor. The overall accuracy of each model's N_2_O performance suggests the single-point simulation to describe the measured data slightly better than the grid-to-grid simulations. However, and importantly, both models predict poorly as negative EF values are commonly found (except at grid point D5), indicating that the mean of the measurements would be a better predictor than either of the models. This result is not unexpected as it can be hugely challenging to predict daily N_2_O emissions due to its inherently high variability.

**Table 4:** Statistical analysis of SPACSYS's performance on dynamics of N2O emissions for grid-to-grid simulations at grid points D5, F3, F5 and G4 only, and the single-point simulation.

Criteria	Total *n* = 158		D5 *n* = 37		F3 *n* = 84		F5 *n* = 19		G4 *n* = 18	
	Grid-to-grid	Single	Grid-to-grid	Single	Grid-to-grid	Single	Grid-to-grid	Single	Grid-to-grid	Single
ME (× 10^− 3^)	0.7	1.0	− 0.5	− 0.6	1.7	2.4	0.3	0.2	− 0.2	2.5
RMSE (× 10^− 3^)	7.0	7.0	7.0	7.0	8.0	7.5	5.0	5.2	4.2	3.0
MAE (× 10^− 3^)	4.0	4.0	4.0	4.0	6.0	5.0	3.1	3.1	2.9	2.6
RE	17.2	3.1	6.7	0.5	− 34.5	− 16.1	5.5	2.5	− 9.9	66.5
EF	− 0.4	− 0.2	0.4	0.4	− 1.3	− 1.0	− 20.2	− 22.5	− 5.3	− 2.2
*r*	0.47	0.49	0.73	0.74	0.26	0.23	0.02	0.03	0.31	0.34
CD	0.62	0.74	0.85	0.84	0.49	0.68	0.05	0.04	0.15	0.42

#### 3.1.4 Herbage biomass

**Fig. 6:**
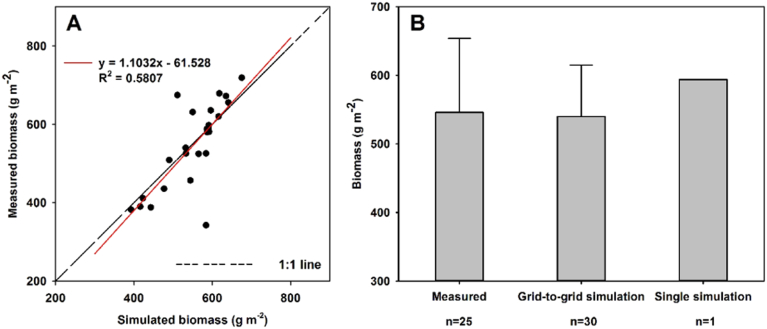
Comparison of measured and simulated ground herbage biomass using the single-point and grid-to-grid simulations.

Grid-to-grid simulations are compared with measured biomass values in the scatterplot (Panel A in Fig. 6), where the measured values ranged from 342 to 719 g m^− 2^. The grid-to-grid simulations tend to agree well with the measured data (Panel B in Fig. 6), with an R^2^ = 0.58, that is clearly adversely influenced by an unusually low biomass prediction of around 580 g m^− 2^ coupled with an unusually high biomass prediction of around 500 g m^− 2^. Observe that a single-point simulation would only provide an average biomass prediction for the whole field. In this respect, predictions would be exactly same at all 25 sites, yielding no spatial detail and a poorer R^2^ value to that found with the grid-to-grid approach.

#### 3.1.5 Summary

The results can be summarised as follows: (A) SPACSYS in both model forms can predict water run-off reasonably accurately, but where there is no advantage to applying the grid-to-grid form over the single-point version; (B) SPACSYS in grid-to-grid form, does not predict soil moisture better than that found from SPACSYS in single-point form, but where both models tend to over-prediction rather than under-prediction; (C) SPACSYS in both model forms poorly predicts N_2_O emissions; (D) SPACSYS in a grid-to-grid form out-performs its single-point version for predicting herbage biomass. Given these results, we choose to only simulate annual soil moisture and biomass at the within-field scale using the grid-to-grid SPACSYS formulation. Soil moisture is chosen given the perceived potential in simulating this data using the grid-to-grid form, as we have not proven an increased accuracy in this respect.

### 3.2 Simulated annual spatial distributions of soil moisture and herbage biomass

#### 3.2.1 Soil moisture

**Fig. 7:**
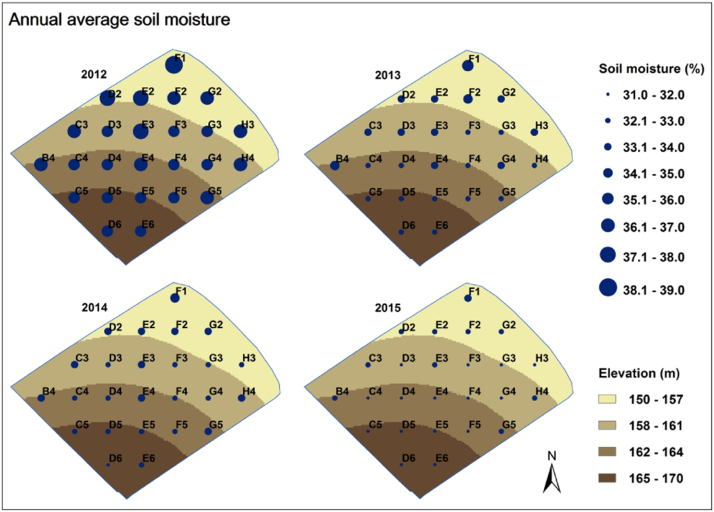
The spatial distributions of simulated annual average soil moisture within Dairy North. The results at the five incomplete grid cells (from Fig. 1) are not shown.

The simulated annual average soil moisture distributions for 2012 through to 2015, at the 25 grid points are shown in Fig. 7. As the field slopes downwards from its southern to northern corners, soil moisture tends to be greater in the north. For example, a soil moisture prediction of nearly 35% is found at grid point F1. In contrast, the driest predictions were at grid points C5, D5, D6 and E6, where elevation is at its lowest. Thus, there is a clear spatial trend in the soil moisture simulations driven by the field's topography. The annual average soil moisture across the field is simulated to range from 35% to 38% in 2012, from 32% to 35% in 2013, from 32% to 35% in 2014, and from 31% to 34% in 2015.

#### 3.2.2 Herbage biomass

**Fig. 8:**
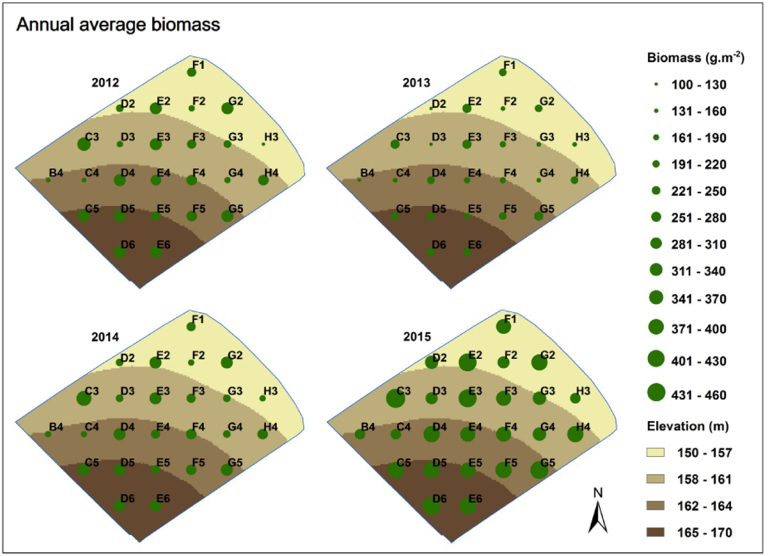
The spatial distributions of simulated annual average ground biomass within Dairy North. The results at the five incomplete grid cells (from Fig. 1) are not shown.

Simulated annual average biomass behaves in a fairly predictable manner, where the spatial characteristics of the simulations are broadly similar year on year, with high levels of predicted biomass at the C3, E2, E3, G2 and G5 grid points, and low levels predicted at the B4, C4, D3, F2 and H3 grid points (Fig. 8). The highest biomass predictions always occur at C3 and E3 grid points, located in the middle of the field, ranging from 225 to 435 g m^− 2^. Simulations across the field ranged from 109 to 330 g m^− 2^ in 2012, from 100 to 249 g m^− 2^ in 2013, from 161 to 342 g m^− 2^ in 2014, and from 256 to 435 g m^− 2^ in 2015.

## 4 Discussion

Clearly model performance is not only dependent on choosing between a single-point and grid-to-grid formulation of the SPACSYS model. There are uncertainties in the input parameters. For example, the soils data only covers six points of a 1.8 ha field, ensuring the grid-to-grid model is not well-informed in this respect and some key parameters are estimated with PTFs. Future work, using higher resolution soils data would likely address this (e.g. Kawamura et al., 2011). There are also issues with the poor spatio-temporal representability of the measured data, especially for the soil moisture and N_2_O model evaluations (which also included long periods of missing data). Future, fully-coherent spatio-temporal sampling campaigns could seek to address this, and in doing so, the grid-to-grid formulation of SPACSYS would be more objectively evaluated. Observe that this study had little to no influence on the collection of measured data sets, as they were often collected for different research purposes than that studied here. The quality of the data collected is however considered high due to stringent quality controls.

For water fluxes, the single-point SPACSYS model can be preferred reflecting the ‘closed system’ nature of this output (i.e., all water run-off drains to a single flume via the NWFP drainage system). There was, however, a certain over-prediction of water discharge, although most measured peak flow events were identified. Water fluxes generated from the grid-to-grid approach also showed less sensitivity to heavy or persistent rainfall events compared to the single-point simulation. This could be due to grid size and water pathways. When an uneven, sloping field is divided into small enough grid cells, the slopes tend to zero, and thus the slope effect on water fluxes can be negligible (Bell et al., 2007, Kuo et al., 1999). Consequently, the grid-to-grid simulation for water fluxes in this study might be relatively un-affected by the slope of the field. At the same time, water flux is modelled ‘immediately’ in the single-point simulations, whilst water flow from the grid-to-grid simulations needs to go through multiple points until finally arriving at the outlet. Hence, this can lead to a smooth flux without the sharp peaks of the measured data. Future work on spatially-adapting the SPACSYS model should investigate further in this respect. Another possible reason to cause the discrepancies between simulated and measured water flux data might be the temporal step. In the model, daily weather data were used in the simulations based on the simulation time step of the model, which could smooth precipitation intensity, and in turn, reduce water fluxes.

For soil moisture, this study cannot provide a definitive result, due to an absence of evaluation data in the spatial dimension. Our study does however, allude to the grid-to-grid formulation providing reasonably accurate soil moisture spatial predictions. Intuitively, these results are promising and are important in that soil moisture inherently varies both spatially and temporally (Longchamps et al., 2015, Shen et al., 2016), where topography can be key drivers of this variation (Gala et al., 2011).

For N_2_O emissions, there was no apparent evidence that the grid-to-grid simulations performed better than the single-point simulations and both approaches performed poorly. Both SPACSYS formulations missed identifying some measured N_2_O emission peaks, whilst they both simulated peaks that were not measured, an effect that has been similarly reported in (Zhang et al., 2016). Some seasonal fluctuations of the measured N_2_O were captured, however. This disappointing model performance can be attributed to many inter-linked factors, one concerns the awkward manner in which the N_2_O emissions were measured, which inherently compromised the model evaluation (as already discussed above). The other mitigating factor is that N_2_O is notoriously difficult to accurately predict, as it is inherently variable both spatially and temporally and at a range of different scales (Chen et al., 2012, DeSimone et al., 2010, Perego et al., 2016, van der Weerden et al., 2014, Xiong et al., 2015). Furthermore, the N_2_O process is dependent on the spatio-temporal processes of soil moisture, temperature and dissolved C (Wu et al., 2016b, Yao et al., 2010), and also on added mineral N by fertilization or organic N from animal excretion, which for the latter is unlikely to be uniformly distributed across the field (Cardenas et al., 2010). Thus, these complex inter-dependencies for N_2_O all need to be reliably accounted for. Future work should seek to develop this.

The SPACSYS model in a grid-to-grid form clearly provides reasonably accurate spatial predictions of plant productivity, yielding an improvement on that found with the single-point, standard model. This outcome is important as spatial variation in herbage biomass is always expected – variation that tends to be associated with heterogeneities in water and nutrients in the soil (Serrano et al., 2016). Herbage biomass is also controlled by field management practices, especially the time, length and number of animals in the field (Kaufmann et al., 2013). Thus, accounting for such spatial characteristics when simulating biomass is an advance.

## 5 Conclusions

This study has demonstrated that a spatially distributed version of SPACSYS can provide a worthy improvement in accuracy over the standard version for biomass productivity. The resulting simulations provide spatial detail, which can help in our understanding of nutrient cycling, water movement and plant growth within a permanent pasture field. No difference in model prediction performance was observed for water run-off, reflecting the closed-system nature of this variable; whilst the simulation results for N_2_O were disappointingly poor, regardless of the SPACSYS formulation used. The soil moisture results with the spatially distributed version could not be objectively verified, but intuitively the simulations appeared promising as they closely followed the study field's topography.

Next steps in this research are to investigate simulating across finer spatial grids for a number of NWFP fields, and to simulate at a higher 15-minute resolution (as the measured NWFP data allow this), rather than the daily resolution considered here. Next steps will also consider the sensitivity of the results to different input and evaluation data sets, where their temporal and spatial resolution needs full consideration. A focus on improving the N_2_O simulations is expected, noting that these simulations are inherently difficult due to this variable's high spatial and temporal variability, with a preponderance to “hot-spots” in both space and time.
